# Unlocking the Antibiofilm Potential of Natural Compounds by Targeting the NADH:quinone Oxidoreductase WrbA

**DOI:** 10.3390/antiox12081612

**Published:** 2023-08-14

**Authors:** Alessandro Ratti, Enrico M. A. Fassi, Fabio Forlani, Maurizio Zangrossi, Matteo Mori, Francesca Cappitelli, Gabriella Roda, Stefania Villa, Federica Villa, Giovanni Grazioso

**Affiliations:** 1 Department of Pharmaceutical Sciences, University of Milan, Via L. Mangiagalli 25, 20133 Milan, Italy; alessandro.ratti@unimi.it (A.R.); enrico.fassi@unimi.it (E.M.A.F.); matteo.mori@unimi.it (M.M.); gabriella.roda@unimi.it (G.R.); stefania.villa@unimi.it (S.V.); giovanni.grazioso@unimi.it (G.G.); 2 Department of Food Environmental and Nutritional Science (DeFENS), University of Milan, Via Celoria 2, 20133 Milan, Italy; maurizio.zangrossi@unimi.it (M.Z.); francesca.cappitelli@unimi.it (F.C.)

**Keywords:** biofilm, WrbA, virtual screening, NADH:quinone oxidoreductase, antioxidant proteins, natural compounds, flavonoids, polyphenols

## Abstract

Biofilm-dwelling cells endure adverse conditions, including oxidative imbalances. The NADH:quinone oxidoreductase enzyme WrbA has a crucial role in the mechanism of action of antibiofilm molecules such as ellagic and salicylic acids. This study aimed to exploit the potential of the WrbA scaffold as a valuable target for identifying antibiofilm compounds at non-lethal concentrations. A three-dimensional computational model, based on the published WrbA structure, was used to screen natural compounds from a virtual library of 800,000 compounds. Fisetin, morin, purpurogallin, NZ028, and NZ034, along with the reference compound ellagic acid, were selected. The antibiofilm effect of the molecules was tested at non-lethal concentrations evaluating the cell-adhesion of wild-type and WrbA-deprived *Escherichia coli* strains through fluorochrome-based microplate assays. It was shown that, except for NZ028, all of the selected molecules exhibited notable antibiofilm effects. Purpurogallin and NZ034 showed excellent antibiofilm performances at the lowest concentration of 0.5 μM, in line with ellagic acid. The observed loss of activity and the level of reactive oxygen species in the mutant strain, along with the correlation with terms contributing to the ligand-binding free energy on WrbA, strongly indicates the WrbA-dependency of purpurogallin and NZ034. Overall, the molecular target WrbA was successfully employed to identify active compounds at non-lethal concentrations, thus revealing, for the first time, the antibiofilm efficacy of purpurogallin and NZ034.

## 1. Introduction

Biofilm is a sessile lifestyle that endows microorganisms with the ability to withstand adverse environmental conditions, thereby contributing to their antimicrobial resistance and persistence in many natural and artificial ecosystems [1]. Biofilms have beneficial effects in microbial biotransformation, soil and water bioremediation, and promoting plant growth [2]. However, the inherent resistance and persistence of biofilms also enhance the virulence of microbial pathogens and hamper their eradication through traditional control treatments [3]. This is the cause of major public health concerns and extends to other fields, such as food safety [4], as well as the control of emerging microbial pests in agriculture [5,6,7].

Currently, antimicrobial agents remain the primary treatment for unwanted biofilms. However, novel strategies are emerging, involving compounds that can interfere with critical stages of biofilm formation. Among these strategies, the investigation of compounds targeting quorum-sensing mechanisms has garnered considerable attention [8,9,10]. A growing body of research has indicated that oxidative imbalance is one of the primary mechanisms that trigger the transition of a microorganism from a planktonic to a biofilm state [11,12,13]. Therefore, oxidative imbalance can serve as a key factor in manipulating the biofilm lifestyle, allowing for either the promotion of or reduction in biofilm formation. As evidence of this concept, the growth of biofilm of *Azotobacter vinelandii* was found to be seven-fold increased in a mutant strain in which chronic oxidative imbalance (i.e., reactive oxygen species (ROS) accumulation) was induced by the deprivation of the antioxidant rhodanese-like protein RhdA [14]. This and other experimental evidence have led to the assessment that chronic sub-lethal oxidative conditions promote the sessile behavior in *A. vinelandii* [14]. On the other hand, the natural compound zosteric acid (ZA) was found to hinder biofilm development at non-lethal concentrations [15,16,17] leading to the accumulation of ROS and the activation of scavenging mechanisms in both bacterial and fungal biofilm model systems [18,19]. The imminent oxidative stress was supposed to act as a cue, prompting bacteria to activate effective scavenging mechanisms or to shift metabolic pathways [20]. The active molecular moiety responsible for the antibiofilm effect of ZA was identified through a structure–activity relationship screening of a 43-member library of molecules that were based on the ZA scaffold [21]. Through a pull-down approach on *E. coli*-soluble proteins, WrbA was identified as the only protein interacting with the active moiety responsible for the antibiofilm effect of ZA [21]. WrbA is an NADH:quinone oxidoreductase enzyme supposed to have a protective role against oxidative stress [22]. The same pull-down approach also revealed the role of WrbA as a molecular target of salicylic acid (SA) [23]. Later, using a similar pull-down approach, WrbA was identified as the protein interacting with the antibiofilm molecule T315, a phenyl-pyrazole analog that targets the etiological agent of typhoid fever, *Salmonella enterica* serovar Typhi [24]. SA, as well as the ZA-analogous compound cinnamic acid (CA), exhibited an antibiofilm effect at non-lethal concentrations [21,23] and demonstrated inhibition of the NADH-dependent oxidoreductase function of *E. coli* WrbA [25,26]. A WrbA-deprived (∆*wrb*A) mutant strain of *E. coli* was used to prove the involvement of WrbA in biofilm formation, which occurs through a mechanism dependent on the ROS level [25]. The lack of WrbA function resulted in a ROS-sensitive phenotype characterized by a reduced number of biofilm-dwelling cells, decreased biofilm thickness, lower matrix polysaccharide content, and reduced tolerance to hydrogen peroxide [25]. The study conducted by Ratti et al. [26] elucidated the underlying mechanisms behind the antibiofilm effect of ellagic acid (EA). For the first time, they highlighted the inhibitory effect of EA on WrbA function, shedding light on the crucial role played by WrbA in mediating the antibiofilm action of EA. The same work revealed a strong correlation between the antibiofilm mode of action of EA and the perturbation of bacterial redox homeostasis driven by WrbA [26]. This mechanism was also proposed to apply to the antibiofilm effect of other compounds such as SA, CA, and ZA [26]. Furthermore, the model proposed by Ratti et al. [26] not only elucidated the mechanisms behind the antibiofilm effect of EA but also shed light on the WrbA-dependency of the antibiofilm compound T315 [24]. Overall, these findings strengthen the importance of WrbA as a promising redox-related target for developing effective antibiofilm agents capable of acting at non-lethal concentrations.

In light of the previous considerations, the main objective of this paper was to harness the potential of the WrbA scaffold as a valuable target for identifying antibiofilm compounds at non-lethal concentrations. The main idea is to hinder biofilm formation by modulating redox homeostasis without killing biofilm-forming microorganisms, but rather by disarming them. This approach seeks to pave the way for innovative strategies that do not rely on biocides. To this purpose, the published three-dimensional structure of WrbA (protein data bank accession code 4YQE, [27]) was exploited to build a computational model suitable for the virtual screening of a library of natural compounds. Subsequently, a selected set of compounds was obtained and subjected to microbiological assays. In particular, the compounds were assessed for their antibiofilm effect at non-lethal concentrations, both individually and in combination, using wild-type and ∆*wrb*A mutant *E. coli* strains. The assessment also included monitoring the levels of ROS experienced by the cells. Finally, the obtained biological data were correlated with terms contributing to the ligand-binding free energy on WrbA to further understand the underlying mechanisms and corroborate the role of WrbA in biofilm modulation.

## 2. Materials and Methods

### 2.1. Virtual Screening of Natural Compounds

The natural compound library was obtained from the ZINC database [28]. To prepare the library for docking, the LigPrep tool of Maestro (LigPrep, Schrödinger, LLC, New York, NY, USA, 2023) was employed to generate corresponding low-energy 3D structures. Filters were applied using the Qikprop tool of Maestro (QikProp, Schrödinger, LLC, New York, NY, USA, 2023) to exclude compounds that lacked drug-like properties. The WrbA binding site was identified by the presence of benzoquinone in the WrbA crystal structure (PDB accession code 4YQE) [27]. The docking grid was centered between flavin-mononucleotide (FMN) and the Trp98, both located within the enzyme’s catalytic site. To enable docking calculations of competitive ligands, benzoquinone was removed from the target structure. To speed up the calculations and to obtain the most reliable results, the compounds were screened using three GLIDE [29] docking calculation steps, with increasing levels of precision. A “high-throughput screening” precision docking calculation was accomplished to remove compounds that did not fit well in the active site of the protein. Then, the 50,000 compounds with the lowest Glide scores were docked using the “Standard precision” (SP) docking protocol. Finally, the top 20,000 compounds from the previous step underwent the extra precision (XP) docking protocol [29]. After visually inspecting the first one thousand compounds ordered by docking score, a hundred WrbA/ligand complexes were built and subjected to molecular dynamics (MD) simulations.

### 2.2. MD Simulations and Binding Free Energy Calculations

The “system builder” tool of Maestro (Schrödinger, LLC, New York, NY, USA, 2023) and the Desmond algorithm [30] were applied to build the simulation system and perform the MD simulations, respectively. The MD simulations time was established considering the stability of the ligands in the catalytic site. For the majority of compounds, 350 ns-long MD simulations were accomplished. To evaluate the ligand stability in the active site, the Cα atoms root mean square deviation (RMSD) vs. time plots were examined. Based on these analyses, the MD 50 frames with the highest ligands stability were considered to calculate the ligand-binding free energy values, using the Molecular Mechanics-Generalized Born Surface Area (MM-GBSA) approach [31,32]. The calculations employed the single trajectory approach, with default parameters of the Prime Maestro protocol. The entropic contribution (∆S) to the ligand-binding free energy (∆G) could not be estimated due to the uncertainties in the calculations and the high computational cost. As a result, we denoted ∆G values as “∆G*”. For this reason, it is not possible to directly translate the calculated ∆G into a Kd value, as ∆G = RT ln Kd. However, these computations can be used as a highly efficient scoring function. Notably, this approach has found successful application in recently published studies [33,34,35,36].

### 2.3. Dataset of Ligand Descriptors for Statistical Analysis

In the subsequent statistical analysis (as described in the following subsection), the ligand–WrbA interaction was integrated by considering all contributions to the ΔG* value of each compound. These contributions encompass various factors, such as electrostatic, hydrogen bond, and van der Waals interactions, which were determined through MM-GBSA calculations (Table S1, Supporting material). The data were extracted from the .csv file generated from the Prime calculations.

### 2.4. Escherichia coli Strains and Growth Condition

The bacterial biofilm model systems used in the study were *Escherichia coli* strains BW25113 and BW25113-∆*wrb*A [25]. The strains were stored in suspensions containing 20% glycerol and 2% peptone at a temperature of −80 °C. For regular cultivation, the strains were grown in Luria-Bertani broth (LB, Sigma-Aldrich/Merck KGaA, Darmstadt, Germany) at a temperature of 30 °C for a duration of 16 h.

### 2.5. Planktonic Growth in the Presence of the Selected Molecules

The ability of *E. coli* strains to grow using the selected compounds as the sole carbon and energy source was tested using phosphate-buffered saline (PBS, Sigma-Aldrich/Merck KGaA, Darmstadt, Germany) supplemented with 500 µM of each molecule. Microbial growth was followed by absorbance at 600 nm (A_600_). All the experiments were repeated three times. The growth of both wild-type and mutant *E. coli* strains in the planktonic state was evaluated in the LB medium supplemented with either 0 μM (positive control) or 500 μM of each selected molecule. The experiments were conducted using 96-well microtiter plates, and growth curves were generated at a temperature of 37 °C using the Infinite^®^ F200 PRO microplate reader (TECAN, Mannedorf, Switzerland). To initiate the experiments, 3 μL (3% *v/v*) of an overnight bacterial culture with a final concentration of 10^6^ cells/mL was added as an inoculum to the wells. The A_600_ was measured at intervals of 10 min over a duration of 24 h. Growth curves were generated by plotting the difference between the A_600_ of the cellular suspensions without and with the molecules and the A_600_ of the non-inoculated medium against the incubation time. The growth curves were fitted using the polynomial Gompertz model using XLSAT software (Version 2022.2.1, Addinsoft, Paris, France). The maximum growth (YM, A_600_) and the x value of the curve inflection point (1/K) were calculated with the same software. Each condition was replicated three times.

### 2.6. Adhesion Assay in the Presence of the Selected Molecules

To evaluate the impact of the selected compounds on cell adhesion, a quantitative analysis was performed using fluorochrome-labeled cells in hydrophobic 96-well black-sided plates, as described in Ratti et al. [26]. Briefly, each well of the microtiter plate contained a final volume of 200 μL of phosphate-buffered saline (PBS) and 10^7^ cells, supplemented with different concentrations (0 μM, 0.5 μM, 5 μM, 50 μM, or 500 μM) of the single molecules. Furthermore, an investigation was conducted to assess the combined effects of the molecules at the lowest concentration of 0.5 μM, in addition to testing the compounds individually. The plates containing both the individual compounds and the combination of molecules were incubated for 18 h at 37 °C. After incubation, the wells were washed twice with 200 μL of PBS, and the adhered cells were subsequently stained with 10 µM SYTO™ 9 (ThermoFisher Scientific, Waltham, MA, USA) in PBS for 20 min at room temperature. The fluorescence intensity was determined using the Infinite^®^ F200 PRO microplate reader (TECAN) with excitation at 483 nm and emission at 503 nm. A standard curve correlating fluorescence intensity with the cell number was generated and employed to quantify the antibiofilm effectiveness of the molecules. The biofilm inhibition efficacy against the wild-type strain was evaluated by calculating the percentage reduction in the number of adhered cells compared to the control groups. The biofilm inhibition efficacy against the ∆*wrb*A mutant strain was evaluated by calculating the percentage reduction in the number of adhered cells compared to the wild-type groups. Six biological replicates were performed for each experimental condition.

### 2.7. Level of Reactive Oxygen Species

The quantification of reactive oxygen species (ROS) in both adhered and non-adhered cells, in the presence or absence of the selected molecules, was performed using the fluorogenic probe CellROX^®^ Green Reagent (ThermoFisher Scientific). The cells were treated with the CellROX^®^ reagent at a final concentration of 5 μM and incubated at room temperature for 30 min. Following incubation, the cells were washed twice with PBS, and the fluorescence intensity was measured using the Infinite^®^ F200 PRO microplate reader (TECAN) at an absorption/emission maxima of 485/520 nm. The fluorescence intensity was normalized by the number of cells. A total of six biological replicates were conducted for each experimental condition.

### 2.8. Statistical Analysis

To determine significant differences among the samples, the T-Test or the analysis of variance (ANOVA) followed by Tukey’s honestly significant difference test (HSD) was performed using the XLSAT software (Version 2022.2.1, Addinsoft). Statistical significance was defined as *p*-values equal to or less than 0.05. To explore the relationship between the biological responses and the terms contributing to the ligand-binding free energy on WrbA (see previous subsection), principal component analysis (PCA) using the XLSTAT software was performed. The correlation matrix was used for PCA, and the first two principal components (PCs) were visually represented in a plot to illustrate the obtained results.

## 3. Results

### 3.1. Virtual Screening of Natural Compounds

Docking calculations were performed to identify natural compounds capable of interacting with the catalytic site of WrbA. For this purpose, a structure-based approach was applied, with *E. coli* WrbA catalytic site serving as the target. A virtual library of 800 k ligands was built using Maestro (Schrödinger LLC, New York, NY, USA) by retrieving compounds from online databases that fulfilled the following criteria: (1) Commercially available, (2) of natural origin, (3) predicted IC50 value for blockage of HERG K+ channels greater than 5.0 µM, (4) predicted logarithm of the octanol/water partition coefficient between −2.0 and 6.5, and (5) predicted logarithm of the aqueous solubility between −6.5 and 0.5 mol/L. The last three filters were applied to the virtual library through QikProp (Schrödinger LLC, New York, NY, USA) calculations (See Material and Methods section for details). Three rounds of docking calculation were conducted, each with increasing precision (see the Material and Methods section for details). The resulting ligands with the most promising scores from the docking calculations were simulated in complex with WrbA to generate a model with the best docking glide score. These complexes were solvated with water molecules and subjected to MD simulations ranging from 300 to 500 ns, depending on the stability of the ligands in the active site of the enzyme. Then, the MM-GBSA approach [31,32] was applied to estimate the ΔG* values of the ligands by analyzing 50 MD frames that represented the ligands with the highest conformational stability in the enzyme’s active site (Table 1). The obtained ΔG* values (Table 1) revealed that several compounds displayed values falling within the range predicted for the reference compounds, ellagic and cinnamic acids [26].

Considering both the compounds with the lowest predicted ΔG* values and their commercial availability and established safety profiles, we identified fisetin, morin, purpurogallin, NZ028, and NZ034 as the prime candidates for further investigation. These compounds, along with EA, were selected for further investigations and purchased from Molport (Molport, SIA, Riga, LV1011, Latvia). Notably, compounds containing sugar moieties and exhibiting high structural similarity to those listed in Table 1 were excluded from further microbiological investigations.

### 3.2. Growth Curves with and without the Selected Compounds

**Figure 1:**
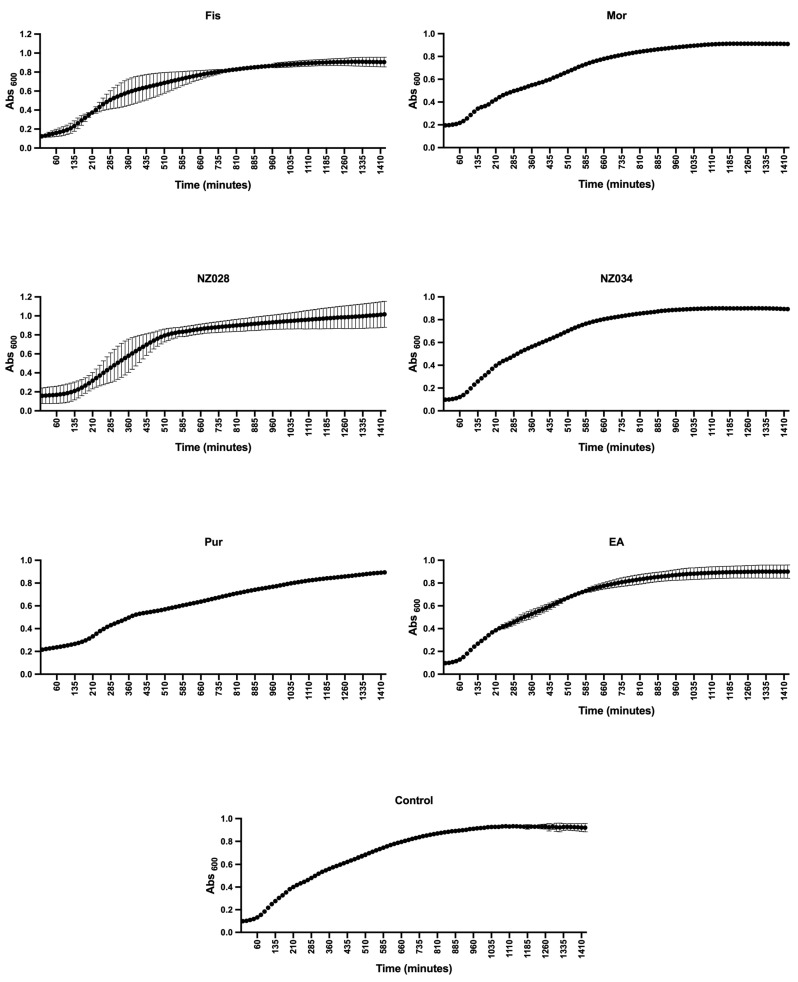
Planktonic growth of *E. coli* wild-type strain with and without (control) the addition of 500 μM of each selected molecule. The data presented represent the mean ± standard deviation obtained from independent measurements.

Before evaluating the antibiofilm properties of each chosen compound, their ability to serve as a carbon and energy source, as well as their potential impact on the planktonic growth of wild-type and WrbA-deprived *E. coli* strains, was investigated. The experiments clearly demonstrated none of the compounds could act as the sole carbon and energy source. This was evident from the absence of growth observed in both bacterial strains when 500 μM of each compound was added to PBS. To assess the influence of the compounds on the planktonic growth of the wild-type and mutant *E. coli* strains, experiments were conducted in a nutrient-rich medium with and without 500 μM of each compound. The growth curves and kinetic parameters obtained from these experiments are presented in Figure 1 and Table 2, respectively. The results indicate that while the maximum growth (YM) remained unchanged in the presence or absence of the compounds, the curve inflection point (1/K) of morin differs from the control (Table 2). However, the 1/K value of morin indicates a higher growth rate compared to the control. Hence, the experimental findings demonstrated that all the selected molecules, at concentrations of up to 500 μM, did not impact the planktonic growth of *E. coli*. Consequently, they did not exhibit any biocidal effects on the cells up to 500 μM.

### 3.3. Antibiofilm Effect of the Selected Compounds and ROS Levels

The antibiofilm performance of the selected compounds at non-lethal concentrations was evaluated against both wild-type and WrbA-deprived *E. coli* strains. The use of the mutant strain enabled the evaluation of WrbA’s role in cell adhesion. The dataset also included the compound EA, which has been proven to rely on WrbA modulation for its antibiofilm activity [26]. For this reason, EA was used as a reference molecule to assess the ability of WrbA to select promising antibiofilm compounds.

**Figure 2:**
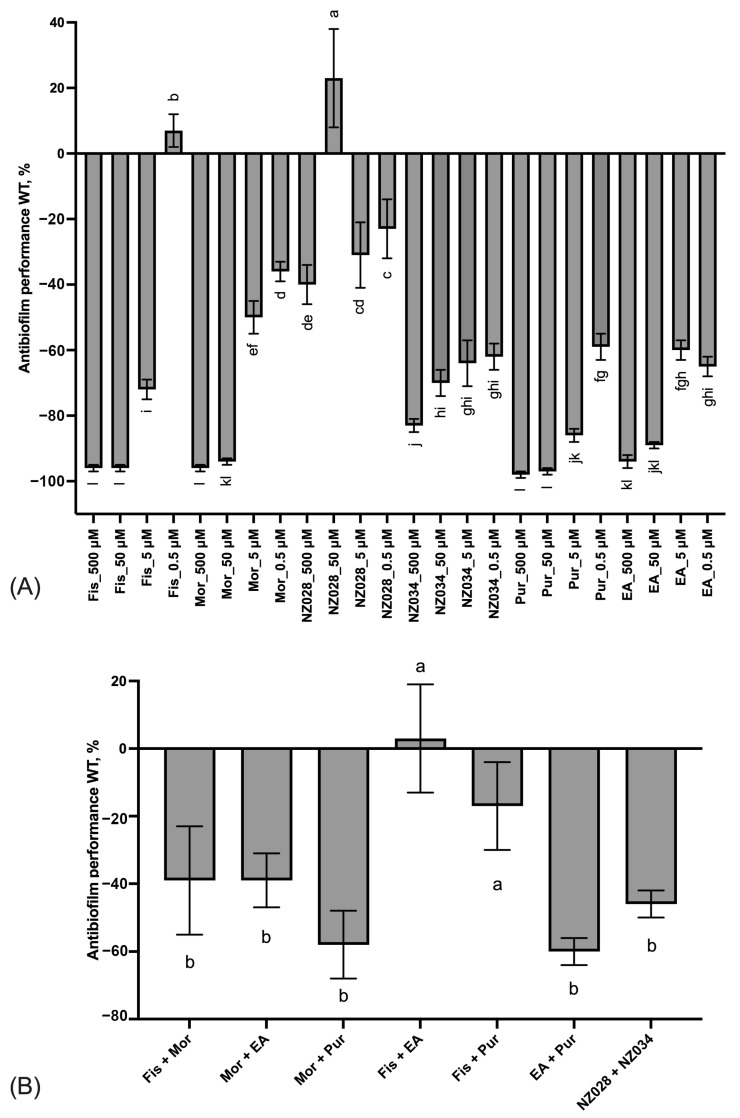
Panel (**A**) displays the antibiofilm effect (%) of the single molecules at different non-lethal concentrations against the *E. coli* wild-type (WT) strain. Panel (**B**) shows the antibiofilm effects (%) of various combinations of the selected molecules at the lowest concentration of 0.5 μM on the WT *E. coli* strain. To quantify the antibiofilm effect, percentage values were calculated by comparing the treated samples with their corresponding control samples. Negative values indicate a reduction in cell adhesion, while positive values a promotion of cell adhesion. The data presented represent the mean ± standard deviation obtained from independent measurements. Different letters indicate statistically significant differences (Tukey’s HSD, *p* ≤ 0.05) between different conditions.

The results of the adhesion assay conducted on the wild-type strain clearly demonstrated a progressive improvement of the antibiofilm effect as the concentration increased (Figure 2A). This trend led to significant inhibition of *E. coli* adhesion, >80%, when treated with 500 μM of fisetin, morin, purpurogallin, and NZ034. Notably, purpurogallin and NZ034 showed excellent antibiofilm effects at the lowest concentration of 0.5 μM, in line with the performance of ellagic acid. Among the tested compounds, NZ028 exhibited the least effective antibiofilm performance across all the concentrations. Specifically, at the highest concentration of 500 μM, NZ028 exhibited a 40% reduction in cell adhesion. Furthermore, the combination of molecules at the lowest concentration of 0.5 μM did not lead to any improvement in the antibiofilm performance compared to using the individual molecules separately (Figure 2B). Among all the combinations tested, the combinations of fisetin with either purpurogallin or ellagic acid resulted in the least favorable antibiofilm effect.

**Figure 3:**
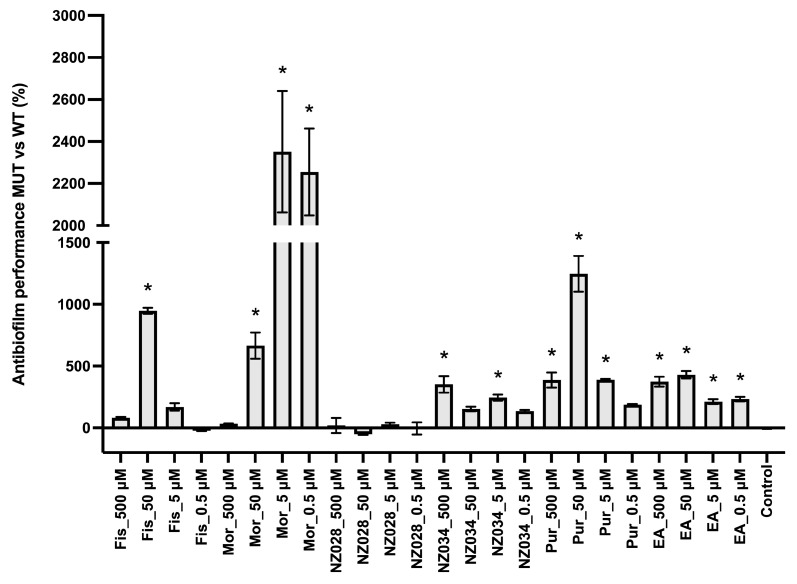
Comparison between the antibiofilm performance of the wild-type (WT) and WrbA-deprived mutant (MUT) strains. To quantify the antibiofilm effect of the molecules against the ∆*wrb*A mutant strain, the percentage decrease (indicated by negative values) or increase (indicated by positive values) in the number of adhered cells compared to the respective WT groups was determined. The data presented represent the mean ± standard deviation obtained from independent measurements. The asterisks indicate statistically significant differences (Tukey’s HSD, *p* ≤ 0.05) from the control samples.

Figure 3 shows the comparison between the antibiofilm performance of the wild-type and the WrbA-deprived mutant strains. Overall, the absence of WrbA negatively impacts the antibiofilm performance of the selected molecules, leading to an increase in the number of adhered cells compared to the experiments conducted with the wild-type strain. Notably, the experiment carried out with the compound NZ028 revealed no statistically significant differences between the treated samples and the control samples.

**Figure 4:**
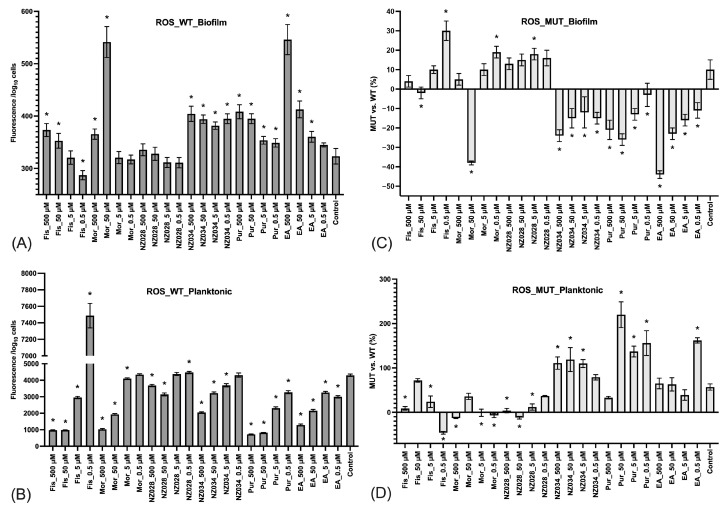
Levels of ROS experienced by the adhered cells (biofilm, panel (**A**)) and non-adherent cells (planktonic, panel (**B**)) of the wild-type *E. coli* strains exposed to different non-lethal concentrations of the selected molecules. The data represent the mean ± standard deviation of independent measurements. Panels (**C**) and (**D**) present a comparison of ROS levels between the wild-type (WT) and the WrbA-deprived mutant (MUT). This comparison was obtained by calculating the percentage decrease (indicated by negative values) or increase (indicated by positive values) in the ROS levels relative to their respective WT groups. The data presented represent the mean ± standard deviation obtained from independent measurements. The asterisks indicate statistically significant differences (Tukey’s HSD, *p* ≤ 0.05) from the control samples.

The ROS level experienced by both adhered (biofilm) and non-adherent (planktonic) cells in the presence or absence of the molecules was also investigated (Figure 4). The results demonstrated that adhered cells of the wild-type *E. coli* strains exhibited low levels of ROS, whereas non-adherent cells experienced high levels of ROS (Figure 4A,B). In addition, the ROS levels in non-adherent cells increased with decreasing concentrations of the molecules (Figure 4B). Furthermore, a comparison was conducted between the ROS levels of the wild-type (WT) and WrbA-deprived mutant (MUT) strains (Figure 4C,D). This comparison was obtained by determining the percentage decrease (denoted by negative values) or increase (denoted by positive values) in the ROS levels compared to their respective WT groups. The mutant strain treated with NZ034, purpurogallin, and EA displayed a decrease in ROS levels in the biofilm-dwelling cells compared to the wild-type strain (Figure 4C). In contrast, the planktonic cells showed higher levels of ROS compared to their respective wild-type groups (Figure 4D).

**Figure 5:**
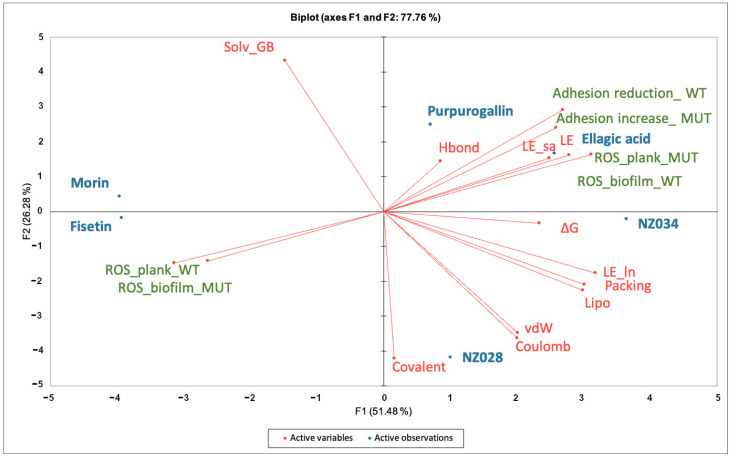
Principal component analyses (PCA) on the data matrix comprising both the microbiological data at the lowest concentration of 0.5 μM and the terms contributing to the ligand-binding free energy on WrbA. The biplot illustrated the distribution of the selected compounds in relation to the characteristic variables.

Principal component analysis (PCA) was conducted on the dataset comprising biological data obtained at the lowest concentration of 0.5 μM and the descriptors of the interactions between the molecules and WrbA (Figure 5). By using the first two PCs, the model accounted for 78% of the variance in the input data matrix. The biplot graph showed a clear distribution of the objects, revealing the presence of three distinct clusters. The first cluster consists of the polyphenols purpurogallin and ellagic acid and the 7-hydroxycoumarin NZ034. This cluster is mainly characterized by the variable “∆G*” and those terms contributing to the ligand-binding free energy on WrbA, such as the ligand efficiency (“LE”) (Table S1, Supporting material). It is worth noting that the molecules within this cluster not only displayed the highest antibiofilm performance but also exhibited a loss of activity in the presence of the ∆*wrb*A mutant strain. This is indicated by the variables “Adhesion reduction_WT” and “Adhesion increase_MUT”, respectively. Ellagic acid, purpurogallin, and NZ034 increased the ROS levels in the planktonic cells of the mutant strain, and this increase in ROS is inversely correlated to the oxidative stress experienced by the biofilm-dwelling cells. The second cluster comprises the 7-hydroxycoumarins NZ028, which is characterized by the terms “Coulomb” and “vdW”, representing the electrostatic and hydrophobic contribution to the ligand-binding free energy values, respectively. These two terms are inversely correlated to the variables “Solv_GB”, indicating the solvation energy contribution to the ligand-binding free energy values. Additionally, no specific biological responses were observed in association with this compound. The third cluster sees the flavonoids morin and fisetin, which are completely unrelated to any of the terms contributing to the ligand-binding free energy on WrbA.

## 4. Discussion

Bacteria have the remarkable ability to colonize and form biofilms on virtually any type of surface, whether natural or synthetic [37,38]. These biofilms have significant implications in many natural and artificial settings, causing chronic illnesses, nosocomial infections, industrial pipe fouling, food spoilage and contamination, plant disease, and even ship-hull fouling [39,40,41,42]. The detrimental effects of biofilms on human society impose a significant societal and economic burden. As a result, there is a growing need for novel strategies that can counteract biofilm formation or mitigate its negative impacts, ideally without exerting a biocidal effect on the biofilm-forming microorganisms. Such approaches can play a crucial role in curbing the spread and development of antimicrobial resistance. The current trend is to use non-lethal antibiofilm strategies able to disarm microorganisms without killing them [43]. Examples of these antibiofilm strategies could include the use of natural-based molecules such as zosteric, salicylic, and ellagic acids, which rely on the enzyme WrbA for their activity [15,23,25,26].

**Figure 6:**
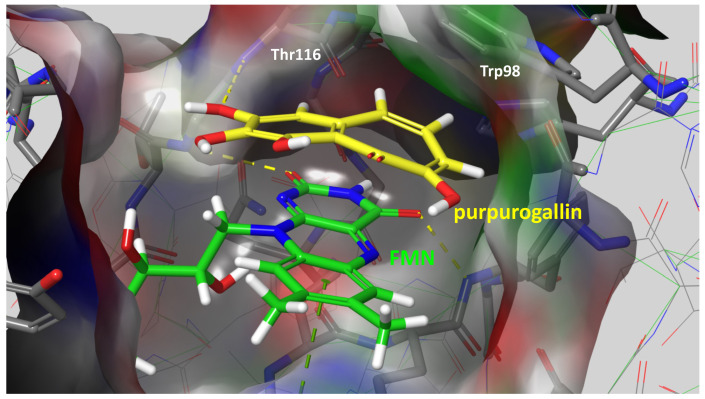
Supposed binding mode of purpurogallin in complex with WrbA. Residues were numbered accordingly with the sequence reported on the Uniprot database (https://www.uniprot.org (accessed on 23 June 2022)), entry code P0A8G6.

Given the compelling evidence of WrbA’s role in biofilm formation [24,25] and the proven antibiofilm properties of molecules interacting with and modulating WrbA’s function, we hypothesized that leveraging the WrbA scaffold could represent a valuable target for selecting antibiofilm compounds at non-lethal concentrations. To achieve our objective, we accomplished a virtual screening of 800,000 natural compounds, using the WrbA scaffold as the target, to identify potential antibiofilm agents that meet the criteria of being commercially available and having safe toxicological profiles. Using the aforementioned computational procedure, five compounds were identified: Fisetin, morin, purpurogallin, NZ028, and NZ034, along with ellagic acid used as a reference molecule. Ellagic acid has been previously shown to rely on WrbA modulation for its antibiofilm activity [26]. Therefore, ellagic acid was used as a benchmark to assess the ability of WrbA in selecting promising antibiofilm compounds. All of the selected compounds displayed binding modes on WrbA that were strictly related to the one predicted for the ellagic acid [26]. In particular, these compounds share the presence of fused planar rings capable of forming π-π stacking with the flavin mononucleotide (FMN) in complex with WrbA. This interaction allows them to effectively occupy the enzyme’s catalytic site. As an example, in Figure 6, the binding mode predicted for purpurogallin is reported. Notably, the seven-member ring of purpurogallin engages in π–π interactions with Trp98 of WrbA, while one of the phenolic OH groups forms a hydrogen bond with the backbone atoms of Thr116.

The flavonoids morin and fisetin were proven to have antibiofilm efficacy at sub-lethal concentrations against *Staphylococcus aureus*, *Streptococcus dysgalactiae*, *Acinetobacter baumannii*, and *Listeria monocytogenes* [44,45,46,47]. Nevertheless, the impact of these compounds on *E. coli* and their reliance on WrbA has not been explored to date.

Before assessing the antibiofilm efficacy of the chosen compounds, their influence on the growth of both the wild-type and mutant strain was examined at the highest concentration of 500 μM. Our findings revealed that the selected molecules did not affect the planktonic bacterial growth up to 500 μM. Therefore, if these compounds demonstrate antibiofilm effects, it would be attributed to a non-lethal mechanism. This is of utmost importance in tackling the emergence of resistant bacterial strains and implementing effective strategies to combat unwanted biofilms [48]. Moreover, it was observed that the tested molecules did not serve as a carbon and energy source at a concentration of 500 μM. Hence, any potential pro-biofilm activity of these compounds should be attributed to mechanisms other than their mere function as growth substrates.

Our findings revealed that, except for NZ028, all of the selected molecules exhibited notable antibiofilm effects. In particular, at the lowest concentration of 0.5 μM, both NZ034 and purpurogallin showed comparable performance to the reference compound ellagic acid. The lower antibiofilm effect of NZ028 compared to NZ034 can be attributed to the different chemical structures of the compound, consequently leading to different physicochemical properties. In fact, the presence of the amino acidic moiety in NZ028, which is absent in NZ034, may potentially affect the antibiofilm performance of the compound. Due to its higher polarity, NZ028 may face challenges in crossing the bacterial cell wall and membrane, thereby hindering its ability to reach the intracellular target WrbA.

The combinations of the different molecules at 0.5 μM were carried out to identify any interaction existing between the individual compounds. Overall, the data suggested antagonistic effects, which occur when the combined effect is less than the expected sum of the two single compounds [49]. It is reasonable to assume that these molecules compete for the same molecular target, reducing the overall efficacy of the compounds in combination [50]. Among all the combinations tested, it was particularly noteworthy that the presence of either purpurogallin or ellagic acid alongside fisetin led to the least desirable antibiofilm effect. Thus, the pro-biofilm effect of 0.5 μM fisetin surpassed the antibiofilm effect of both purpurogallin and ellagic acid. It is possible that these compounds act as either antioxidant or pro-oxidant species depending on the concentration [13]. At a low concentration, the presence of fisetin may have counteracted the activity of purpurogallin and ellagic acid, possibly through an antioxidative interaction. The antioxidant properties of fisetin might have offset the ROS imbalance induced by purpurogallin and ellagic acid, thereby reducing their effectiveness in inhibiting biofilm formation.

The absence of WrbA had a significant impact on the overall antibiofilm performance of the selected molecules, reducing the antibiofilm effectiveness and increasing the number of adhered cells compared to the experiments conducted with the wild-type strain. This was particularly evident in the case of purpurogallin, ellagic acid, and NZ034, as they consistently exhibited increased biofilm biomass in the mutant strain across all tested concentrations. The pro-biofilm effect of the molecules on the mutant strain was further associated with a reduction in ROS levels experienced by the adhered cells, as compared to the wild-type strain. In contrast, the non-adherent cells of the mutant strain demonstrated a higher level of ROS. WrbA is an enzyme that plays a crucial role in cellular defense against oxidative stress [22,25]. At sub-lethal concentrations, certain molecules, such as polyphenolic substances and coumarins, are believed to function as pro-oxidants by disrupting the normal redox cycle, leading to an accumulation of ROS [13,51]. The lack of protective effect of WrbA may cause a redox imbalance, which generates the driving force for biofilm formation [26]. Several studies reported that oxidative stress can effectively stimulate the growth of biofilms [52,53]. Indeed, biofilm-dwelling cells are able not only to withstand oxidative stress but also utilize it as an advantage. Biofilm-dwelling cells can perceive certain ROS levels as signals or cues, enabling them to prime themselves for adaptation to environmental changes [20]. Furthermore, biofilm-dwelling cells exhibit enhanced tolerance to oxidative pressure, consistently maintaining their redox equilibrium [53]. Bacterial cells possess robust mechanisms to respond to oxidative stress, activating diverse defense systems that have been shown to be highly effective [54]. In a recent study by [25], it was reported that a mature biofilm formed by a WrbA-deprived *E. coli* mutant strain experienced lower oxidative stress compared to a young biofilm, due to the activation of catalases. In addition, the comparative proteomic study on *E. coli* planktonic cells exposed to ZA indicated the promotion of the expression of various stress-response proteins to escape from the oxidative stress experienced by the treated cells [18].

A PCA analysis was conducted to establish a correlation between the antibiofilm performance of the chosen molecules and their dependency on WrbA. The biplot clearly indicated the clustering of purpurogallin, ellagic acid, and NZ034. These molecules not only exhibited promising antibiofilm activity at low concentrations but also displayed reduced efficacy in the presence of the mutant strain. Additionally, based on MM-BSA calculations that describe the ligand–WrbA interactions, these compounds exhibited the highest predicted affinity towards WrbA. As shown in Figure 5, the majority of the terms contributing to ∆G* values of the compounds were clustered in the same PCA area where the antibiofilm and antioxidant properties of purpurogallin, ellagic acid, and NZ034 were also located. Altogether, these findings strongly suggest that the antibiofilm activity of purpurogallin, ellagic acid, and NZ034 is dependent on WrbA. In contrast, the results from the microbiological investigations, along with their relative location in the biplot, suggested that the flavonoids morin and fisetin are less reliant on the presence of WrbA for their antibiofilm mechanism of action when compared to purpurogallin, ellagic acid, and NZ034. Hence, the antibiofilm efficacy of fisetin and morin was likely attributed to their interactions with multiple protein targets, exhibiting varying degrees of binding affinity. A recent study conducted by Chemmugil et al. [46] revealed that morin interacts with the global regulatory protein SarA of *Staphylococcus aureus*. SarA is a central regulatory element that controls biofilm formation and virulence factors production in *S. aureus* [55,56]. The interaction between morin and SarA strongly suggests the potential of morin to act as an effective anti-quorum sensing agent [46]. Through in silico molecular docking analysis, it was observed that the binding efficacy of flavonoids (including fisetin) with the biofilm master regulator BfmR was associated with their antibiofilm efficacy [47]. In *A. baumannii,* BfmR controls genes involved in cell attachment to abiotic surfaces and regulates the chaperone–usher assembly system (*csuA/BABCDE*), which controls pilin production and cell motility machinery [57].

## 5. Conclusions

In this study, the molecular target WrbA was employed to investigate the potential of five natural compounds, namely fisetin, morin, purpurogallin, NZ028, and NZ034, as promising antibiofilm agents capable of modulating cell redox homeostasis. It was shown that, except for NZ028, all of the selected molecules exhibited promising antibiofilm effects at non-lethal concentrations. Notably, at the lowest concentration of 0.5 μM, both NZ034 and purpurogallin showed comparable performance to the reference compound ellagic acid.

The correlation observed between the response of the WrbA-deprived *E. coli* strain and the terms characterizing the interaction between the molecules and WrbA strongly suggests the dependence of purpurogallin and NZ034 on WrbA. However, it is important to acknowledge that WrbA may serve as just one of the molecular targets for the investigated molecules, as evident in the case of fisetin and morin.

In summary, the molecular target WrbA was successfully employed for the identification of antibiofilm compounds at non-lethal concentrations, revealing, for the first time, the efficacy of purpurogallin and NZ034. Leveraging the potential of WrbA as a target for selecting antibiofilm compounds holds great significance in the ongoing battle against unwanted biofilms in many natural and industrial settings. It enables the development of novel biocide-free strategies that specifically hinder biofilm formation without killing microbial cells. This approach not only maximizes the effectiveness of antibiofilm treatments but also promotes sustainability by reducing the reliance on broad-spectrum antimicrobials.

## Figures and Tables

**Table 1:** Chemical structure and predicted ΔG* values of the natural binder of WrbA as determined by virtual screening studies. Among the compounds, the ones highlighted in bold were specifically chosen for further microbiological investigations.

Compounds	Structure	ΔG (SD) Kcal/mol
Ellagic acid, EA (reference)	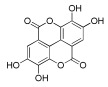	−70.3 ± 4.7
Cinnamic acid (reference)	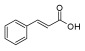	−31.5 ± 2.3
ZINC14641245 Urolithin M5	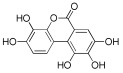	−67.1 ± 7.7
**ZINC13132551** **Purpurogallin, Pur**	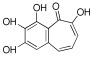	**−52.2 ± 6.2**
ZINC6070307 Aurantio-obtusin	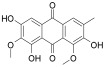	−66.7 ± 10.9
ZINC4098644 Aloe emodin	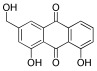	−65.7 ± 4.0
ZINC4098704 Rheina	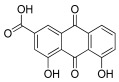	−60.4 ± 5.4
ZINC6536302 Isoscutellarein	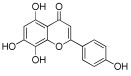	−62.0 ± 5.2
**ZINC39111** **Fisetin, Fis**	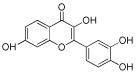	**−48.2 ± 5.0**
ZINC3870337 (−) Gallocatechin	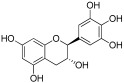	−48.1 ± 7.7
**ZINC3881558** **Morin, Mor**	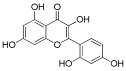	**−40.0 ± 3.2**
ZINC1075952383	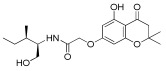	−58.2 ± 4.1
**NZ028**	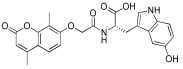	**−85.6 ± 3.1**
**NZ034**	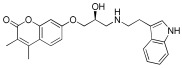	**−80.6 ± 5.0**

**Table 2:** The growth parameters 1/K (representing the x value of the curve inflection point) and YM (indicating the maximum growth) were determined using the Gompertz model applied to the planktonic growth curves of *E. coli*, both with and without 500 μM of each selected molecule. The goodness of fit (R^2^) was also calculated to assess the quality of the Gompertz models. The data presented in the table represent the mean values ± SDs obtained from independent measurements. Statistically significant differences between conditions were determined using Tukey’s honestly significant difference test (HSD) with a significance level set at *p* ≤ 0.05. Different superscript letters were used to indicate significant differences between conditions.

	Fis	Mor	NZ028	NZ034	Pur	EA	Control	*p*-Value
YM	0.926 ± 0.07	0.943 ± 0.02	0.919 ± 0.14	0.909 ± 0.001	0.950 ± 0.004	0.913 ± 0.06	0.947 ± 0.02	0.673
1/K	200.5 ± 22.4 ^a^	157.5 ± 7.8 ^b^	272.1 ± 23 ^ab^	189.7 ± 2.2 ^ab^	206.5 ± 3.1 ^a^	197.4 ± 9.8 ^a^	197 ± 10.2 ^a^	0.005
R^2^	0.9488	0.9960	0.9121	0.9970	0.9939	0.9792	0.9942	

## Data Availability

All of the data is contained within the article and the supplementary materials.

## Supplementary Materials

The following supporting information can be downloaded at: https://www.mdpi.com/article/10.3390/antiox12081612/s1, Table S1: MM-GBSA terms used for PCA.
