# Supplementary material for: Unlocking the Antibiofilm Potential of Natural Compounds by Targeting the NADH:quinone Oxidoreductase WrbA
Source: Antioxidants (Basel). 2023 Aug 14;12(8):1612. doi: 10.3390/antiox12081612 (PMC10451263; doi:10.3390/antiox12081612)
Supplement: Supplementary file 1 [file antioxidants-12-01612-s001.zip › Supporting_material.pdf]

# *Supporting material for*

# Unlocking the Antibiofilm Potential of Natural Compounds by Targeting the NADH:quinone Oxidoreductase WrbA

**Alessandro Ratti[1], Enrico M. A. Fassi[1], Fabio Forlani[2,*], Maurizio Zangrossi[2], Matteo Mori[1], Francesca Cappitelli[2], Gabriella Roda[1], Stefania Villa[1], Federica Villa[2,*], and Giovanni Grazioso[1,*]**

**Table S1.** MM-GBSA terms resulted in the output of the Prime calculations and then used for PCA. The abbreviation used in the mnuascript (Figure 5) are highligted in bold.

| **Term** | **Description** |
|---|---|
| r_psp_MMGBSA_dG_Bind(NS)_**Coulomb** | Coulomb energy |
| r_psp_MMGBSA_dG_Bind(NS)_**Covalent** | Covalent binding energy |
| r_psp_MMGBSA_dG_Bind(NS)_**Hbond** | Hydrogen-bonding energy |
| r_psp_MMGBSA_dG_Bind(NS)_**Lipo** | Lipophilic energy |
| r_psp_MMGBSA_dG_Bind(NS)_**Packing** | Pi-pi packing energy |
| r_psp_MMGBSA_dG_Bind(NS)_**Solv_GB** | Generalized-Born electrostatic solvation energy |
| r_psp_MMGBSA_dG_Bind(NS)_**vdW** | Van der Waals energy |
| TOTAL (**ΔG**) | Binding free energy value |
| r_psp_Prime_MMGBSA_ligand_efficiency (**LE**) | ΔG / (number of heavy atoms) |
| r_psp_Prime_MMGBSA_ligand_efficiency_sa (**LE_sa**) | ΔG / (number of heavy atoms)$^{2/3}$ *This efficiency metric approximates the effect of surface area* |
| r_psp_Prime_MMGBSA_ligand_efficiency_ln (**LE_ln**) | ΔG / (1 + ln(number of heavy atoms)) |
